# Gastric Outlet Obstruction Caused by Acute Gastric Volvulus: A Rare Complication of Hiatal Hernia

**DOI:** 10.7759/cureus.38609

**Published:** 2023-05-05

**Authors:** Harshavardhan Sanekommu, Zaid Siddiqui, Aidan Farrell, Sobaan Taj, Arif B Saleh, Ennis Alsaadi, Pranav Shah

**Affiliations:** 1 Internal Medicine, Jersey Shore University Medical Center, Neptune City, USA; 2 Internal Medicine, St. George's University School of Medicine, Saint George's, GRD; 3 Internal Medicine, Hackensack Meridian School of Medicine, Nutley, USA; 4 Radiology, Jersey Shore University Medical Center, Neptune City, USA

**Keywords:** robot-assisted nissen fundoplication, gerd, gastric outlet obstruction, haital hernia, gastric volvulus

## Abstract

Hiatal hernias are commonly encountered in elderly patients, predisposing patients to the common condition of gastroesophageal reflux disease (GERD). Depending on the size of the hernia, different complications can arise. Large hernias can lead to development of gastric volvulus, obstruction, strangulation, and perforation. Therefore, management of large hiatal hernias is crucial to avoid such complications. In this paper, we describe a patient who presented with acute gastric volvulus secondary to a large hiatal hernia. She improved with conservative management and subsequently underwent successful repair of the hernia. We emphasized the importance of identifying gastric volvulus among its vague presentation for prompt management.

## Introduction

Acute gastric volvulus is an uncommon, but life-threatening pathology, where the stomach is rotated beyond 180 degrees. Gastric volvulus is difficult to diagnose due to nonspecific symptoms that are often mistaken for common causes of abdominal pain [1]. Symptoms also depend on the speed of onset, the type of volvulus, and the degree of obstruction, with patients presenting with pain, severe retching, and inability to pass a nasogastric tube (Borchardt’s triad) in the acute form, and nonspecific symptoms in the chronic form [1]. Complications include strangulation leading to necrosis and potential perforation [2]. Though standalone gastric volvulus is rare, an even less common presentation is caused by a comorbid hiatal hernia [3]. We present a 79-year-old female patient with this condition to highlight the importance of maintaining clinical suspicion of acute gastric volvulus in patients with nonspecific abdominal symptoms.

## Case presentation

**Figure 1:**
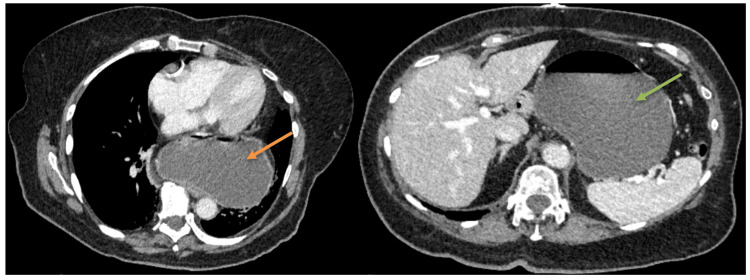
A large gastric hiatal hernia (orange arrow) with marked gastric distention and fluid retention (green arrow) suggesting gastric outlet obstruction.

A 79-year-old female patient with a past medical history of hiatal hernia for the past five years, gastroesophageal reflux disease (GERD), and coronary artery disease presented to the emergency department with complaints of severe epigastric pain, nausea, and nonbilious/nonbloody vomiting. The pain started after having lunch the day prior and at night time, she started to experience vomiting. She has been on chronic proton pump inhibitor (PPI) therapy for her GERD. On arrival, the patient's vitals were as follows: blood pressure 148/96 mmHg, heart rate 98 beats per minute, temperature 98°F, respiratory rate 18 breaths per minute, and oxygen saturation of 97% on room air. Physical examination was only significant for tenderness in the epigastric region with diffuse tympanic hyperresonant throughout. Significant lab values were as follows: white blood cell count 12.8/mm³, glucose 212 mg/dL, blood urea nitrogen 14 mg/dL, creatinine 0.8 mg/dL, lipase 79 U/L, and lactic acid 2.1 mmol/L. Computed tomography (CT) of the abdomen and pelvis with contrast revealed a large paraesophageal hernia with marked gastric distention and fluid retention - suggestive of gastric outlet obstruction (GOO) (Figure 1).

**Figure 2:**
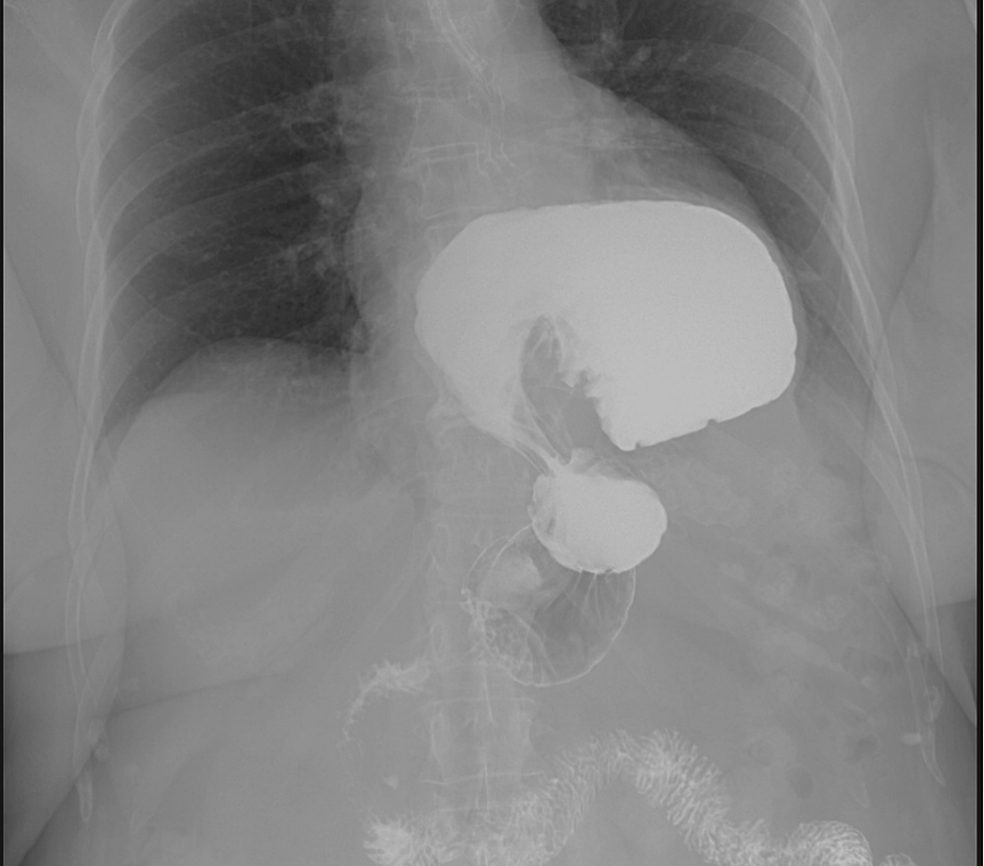
Upper GI series showing a large paraesophageal hernia with organoaxial gastric volvulus with contrast seen within the small bowel.

The patient was managed conservatively with intravenous (IV) fluids and nasogastric tube placement for decompression. The patient also underwent an upper gastrointestinal (UGI) series which also revealed a large paraesophageal hernia and a resolving gastric outlet obstruction from gastric volvulus (Figure 2).

Two days into her hospital stay, the patient’s nasogastric (NG) tube was removed due to a fully resolved gastric volvulus. A day after, she successfully underwent a robot-assisted hiatal hernia repair with anterior 180° fundoplication. Postoperative course was unremarkable and the patient was discharged after a day of observation. In a three-month follow-up, patient’s symptoms of gastroesophageal reflux disease (GERD) have greatly reduced and she is able to eat a wider variety of foods.

## Discussion

Hiatal hernias or paraesophageal hernias are relatively common and increase in prevalence with age, affecting up to 60% of the population above the age of 50 years [4]. Rarely, large hiatal hernias can result in malrotation of the stomach leading to the development of a gastric volvulus [1,4]. We present a case of an elderly woman with a large hiatal hernia who subsequently developed gastric volvulus, which was resolved by conservative management.

Hiatal hernias can develop due to defects in the esophageal hiatus, which may be congenital or acquired. As the hernia enlarges, the stomach may protrude through the diaphragm into the mediastinum, causing complications such as GERD, incarceration of the bowels, and gastric volvulus [4]. Large hiatal hernias are more likely to result in severe complications, with gastric volvulus being one of the most serious. This condition is often associated with other underlying conditions such as adhesions or hiatal hernias [5].

The typical symptoms of acute gastric volvulus are known as Borchardt’s triad, which consists of nonproductive vomiting, severe epigastric pain, and issues with insertion of a nasogastric tube [5]. In the case of our patient, she had one of the three typical symptoms of Borchardt’s triad. A possible explanation for this atypical presentation is that the patient was at higher risk of chronic gastric volvulus, with a preexisting large hiatal hernia. In these circumstances, the gastric volvulus does spontaneously self-resolve [6]. In the upper GI series, it is already showing signs of resolvement of GOO as contrast was seen seeping into the duodenum. The varied presentation of gastric volvulus adds an extra layer of difficulty in accurately identifying it. In the case presented by Imperatore et al., the patient presented with symptoms of chest pain [4]. Their patient’s volvulus was discovered on CT but due to differing circumstances and presentation, the patient passed away [5]. In the case presented by Lourenço et al., their patient presented similarly to ours. However, their patient presented over the course of 10 days, whereas our patient’s symptoms presented a day prior to admission [2].

The treatment of gastric volvulus has evolved in recent decades, with immediate insertion of a nasogastric tube to facilitate decompression and surgical consultation recommended upon diagnosis to mitigate risks associated with vascular compromise and death [4]. Operative intervention is generally planned, although conservative approaches have been successful in certain cases [3]. In our patient, conservative management was enough for the resolution of gastric volvulus. The focus shifted to prevention of recurrence, therefore, she underwent repair of the hiatal hernia, which was her greatest risk factor.

The management of hiatal hernias typically is determined by the severity. Minor hiatal hernias that are symptomatic can generally be managed with PPIs. More severe hernias require surgical approach [4]. Modern surgical treatment of hiatal hernias can be done laparoscopically with robotic assistance. Robotic assistance allows for comparable outcomes to non-robotic surgeries, however, surgeons have increased ease and dexterity which can result in a longer-lasting correction [7]. Our patient was treated with a robot-assisted Nissen fundoplication that helped not only to reduce the risk of recurrent volvulus but also aided in reducing her GERD symptoms. In a retrospective study, GERD was cured in 98.4% of patients three months after Nissen fundoplication surgery [8].

## Conclusions

In conclusion, acute gastric volvulus is a rare but life-threatening complication of hiatal hernias. In patients with vague abdominal symptoms, it is important to consider gastric volvulus as a differential and to be prepared for prompt medical and surgical treatment. This case illustrates the effectiveness of robot-assisted Nissen fundoplication in treating large hiatal hernias and their complications, which include gastric volvulus and symptoms of GERD.
